# A novel copper complex induces ROS generation in doxorubicin resistant Ehrlich ascitis carcinoma cells and increases activity of antioxidant enzymes in vital organs in vivo

**DOI:** 10.1186/1471-2407-6-267

**Published:** 2006-11-15

**Authors:** Ananda Mookerjee, Jayati Mookerjee Basu, Surajit Majumder, Shilpak Chatterjee, Gouri S Panda, Pranabananda Dutta, Smarajit Pal, Pratima Mukherjee, Thomas Efferth, Syamal Roy, Soumitra K Choudhuri

**Affiliations:** 1 Department of Environmental Carcinogenesis and Toxicology, Chittaranjan National Cancer Institute (CNCI), 37 S. P. Mukherjee Road, Calcutta-700026, India; 2 Department of Immunology, Indian Institute of Chemical Biology, Calcutta– 700 032, India; 3 Department of Clinical Biochemistry, Hospital Unit, CNCI, 37 S. P. Mukherjee Road, Calcutta-700026, India; 4 Department of In-vitro Carcinogenesis and Cellular Chemotherapy, CNCI, 37 S. P. Mukherjee Road, Calcutta-700026, India; 5 German Cancer Research Center, Heidelberg, Germany

## Abstract

**Background:**

In search of a suitable GSH-depleting agent, a novel copper complex viz., copper *N*-(2-hydroxyacetophenone) glycinate (CuNG) has been synthesized, which was initially found to be a potential resistance modifying agent and later found to be an immunomodulator in mice model in different doses. The objective of the present work was to decipher the effect of CuNG on reactive oxygen species (ROS) generation and antioxidant enzymes in normal and doxorubicin-resistant Ehrlich ascites carcinoma (EAC/Dox)-bearing Swiss albino mice.

**Methods:**

The effect of CuNG has been studied on ROS generation, multidrug resistance-associated protein1 (MRP1) expression and on activities of superoxide dismutase (SOD), catalase (CAT) and glutathione peroxidase (GPx).

**Results:**

CuNG increased ROS generation and reduced MRP1 expression in EAC/Dox cells while only temporarily depleted glutathione (GSH) within 2 h in heart, kidney, liver and lung of EAC/Dox bearing mice, which were restored within 24 h. The level of liver Cu was observed to be inversely proportional to the level of GSH. Moreover, CuNG modulated SOD, CAT and GPx in different organs and thereby reduced oxidative stress. Thus nontoxic dose of CuNG may be utilized to reduce MRP1 expression and thus sensitize EAC/Dox cells to standard chemotherapy. Moreover, CuNG modulated SOD, CAT and and GPx activities to reduce oxidative stress in some vital organs of EAC/Dox bearing mice. CuNG treatment also helped to recover liver and renal function in EAC/Dox bearing mice.

**Conclusion:**

Based on our studies, we conclude that CuNG may be a promising candidate to sensitize drug resistant cancers in the clinic.

## Background

Oxidative stress is linked to carcinogenesis as well as to sensitivity or resistance of cancer cells to anticancer drugs [1]. The involvement of reactive oxygen species (ROS) in induction of apoptosis of various cancer cells, especially drug resistant cancers is well known [2-4]. Often, the ability of a therapeutic agent to induce apoptosis of cancer cells depends upon the ability of cancer cells to generate ROS [5]. Moreover, low levels of ROS favor the expression of ABC transporters like P-gp [6]. Drug resistant cancers often show very low levels of ROS. This is usually due to high intracellular reduced glutathione (GSH) levels [7,8] and enhanced activities of antioxidant enzymes like glutathione peroxidase (GPx), catalase (CAT) and superoxide dismutase (SOD) [9]. On the other hand, GSH is also required for phase II detoxification reactions, for example phase II enzymes like glutathione S-transferase (GST) isozymes require GSH for the conjugation of electrophilic drugs and xenobiotics [10]. Therefore, high levels of GSH and GST have been implicated in drug resistant tumors [8,11,12].

It has been reported that the depletion of GSH by different pharmacological agent modulates resistance to anticancer drugs [8,13,14]. So far no systematic approach of lowering GSH levels has been made, and the chemicals capable of lowering GSH level and GST activity have no structure-activity relationships [13,14]. Unfortunately, most compounds lowering GSH are toxic at required doses [13,14]. Therefore, the search for compounds having high GSH-depleting properties with low toxicity is of immense importance in the field of reversal of multidrug resistance.

For this reason, we have developed a novel metal chelate capable of depleting GSH at non-toxic doses. Previously, we have reported the synthesis, toxicity and resistance reversal activity of the complex, viz., copper *N*-(2-hydroxyacetophenone) glycinate (CuNG) [15,16]. A single administration of CuNG can overcome multidrug resistance (MDR) in vivo in male Swiss albino mice bearing doxorubicin (dox) resistant Ehrlich ascites carcinoma (EAC/Dox) cells; CuNG in combination with dox increases the survivality (~4.5 folds with respect to untreated control) [16]. Moreover, CuNG treatment alone at a lower dose (single administration) was found to completely heal EAC/Dox bearing animals from their tumor through immunomodulation in vivo [17]. Since copper has been reported by others to induce apoptosis by generation of ROS [18], and since CuNG is a copper(II) chelate, it warranted a study on its effect on ROS generation, which includes beside GSH, a number of antioxidant enzymes, like GPx, SOD and CAT.

In the present investigation we report that in vivo treatment of novel metal chelate (CuNG) in one hand induced ROS and down-regulated surface multidrug resistance-associated protein 1 (MRP1) expression in EAC/Dox cells, and on the other hand increased activities of antioxidative enzymes in vital organs like heart, lung and kidney which might be involved in CuNG mediated decrease in ROS levels in those organs. Moreover, CuNG got excreted through urine and bile, thus rendering animals safe from copper toxicity.

## Methods

### Materials

Reduced glutathione (GSH), 5,5'-dithio bis (2-nitrobenzoic acid) (DTNB), Nitro Blue Tetrazolium (NBT) and catalase were purchased from Sigma Chemical Company, St. Louis, USA. Ortho dianisidine and xanthene oxidase were purchased from Acros Organics, Geel, Belgium. Other chemicals used were of highest purity available. Anti-MRP1 polyclonal antibody was purchased from Santa Cruz Biotech. Inc (SC).

### Animals and Cell lines

Swiss albino mice, originally obtained from National Institute of Nutrition, Hyderabad, India and reared in the institute animal facilities, were used for all in vivo experiments with prior approval of the institutional animal ethics committee. Dox resistant Ehrlich ascites carcinoma (EAC/Dox), which is also resistant against cisplatin, cyclophosphamide and vinblastin [14] was developed and maintained according to the methods described previously [14]. In brief, 1 × 10^6 ^EAC/Dox cells were inoculated intraperitoneally (i.p.) into each mouse (weighing 18–22 gm, 6 weeks old) of experimental group(s) and maintained as an ascitic tumor. During experiments, ascitic fluid containing EAC/Dox cells was drawn out aseptically from peritoneal cavity of mice having 7–8 days of ascitic tumor growth. Tumor cells were washed in phosphate buffer saline (PBS, pH 7.4) and counted in a haemocytometer by trypan blue exclusion method.

### Treatment schedule of CuNG and Dox

CuNG was injected once i.p. at a dose of 10 mg/kg body weight [dissolved in DMSO and finally suspended in normal saline to achieve final concentration of 0.1% (v/v) DMSO in normal saline]. Untreated control group received i.p. injection of 1 ml vehicle (i.e., 0.1% (v/v) DMSO in normal saline) only. In EAC/Dox bearing mice, CuNG was administered 7 days after inoculation with EAC/Dox. Previous work from our laboratory had showed that at a dose of 10 mg/kg body weight CuNG had no toxicity [15]. Animals were euthanasized 2 h or 24 h after CuNG treatment unless mentioned otherwise.

### Measurement of glutathione (GSH)

GSH was measured following the method of Sedlack and Lindsay [19]. Briefly, tissue/1 × 10^6 ^cell homogenate in 0.1 ml PBS was mixed with 2.4 ml EDTA (0.02 M), 2 ml deionized water and 0.5 ml 50% TCA and centrifuged at 500 × g for 15 min at 4°C. 2 ml of supernatant was mixed with 2 ml 0.4 M Tris buffer (pH 8.9). 50 *μl *5,5'-dithio bis (2-nitrobenzoic acid) [DTNB] (0.01 M) was added to the mixture. Within 2–3 min of addition of DTNB, optical density (OD) was measured at 412 nm. Protein was measured by using the Bradford Method [20].

### Measurement of glutathione peroxidase (GPx) activity

GPx activity was measured from the tissue homogenate. Tissue homogenate was prepared following the method of Hafemann *et al *[21]. In brief, the animals were sacrificed, the organs were dissected, dried and weighed. The homogenate was prepared with 0.15 M KCl solution and centrifuged at 10,000 rpm for 20 min at 4°C. The supernatant was analysed according to the reported method [21,22].

### Measurement of catalase (CAT)

Catalase was measured by the reported method [21,23]. In brief, tissue homogenate was prepared using 0.1 M phosphate buffer solution (PBS, pH7.4) and centrifuged at 1,00,000 g for 1 h at 4°C. Tissue homogenate was transferred in 0.1 M PBS (pH 7.4) containing 0.45 M H_2_O_2. _Aliquots of the mixture (0.5 ml) were removed at 20 S intervals and added to 2.0 ml of solutions containing 0.2 mg/ml O-dianisidine, 0.015 mg/ml peroxidase and 0.81 mg/ml sodium azide. After 10-min incubation at room temperature, 50% H_2_SO_4 _solution was added to stop the reaction. The absorbance of the reaction mixture was measured at 530 nm. One unit of enzyme activity (k) was calculated as follows:

k' = (2.303/t) log [a/(a-x)] [a, starting conc. of H_2_O_2_; a-x, H_2_O_2 _conc. after t time].

### Measurement of Superoxide dismutase (SOD)

SOD was measured by the reported method [21,24]. In brief, tissue homogenate was prepared using 0.1 M PBS (pH 7.4) and centrifuged at 1,00,000 × g for 1 h at 4°C. The supernatant was dialyzed overnight against 0.1 M PBS (pH 7.4) and transferred to a reaction mixture containing 0.043 M Na_2_CO_3 _buffer (pH 10.2), 0.1 mM xanthine, 0.1 mM EDTA, 0.05 mg/ml bovine serum albumin, 0.025 mM nitro blue tetrazolium (NBT) and the sample. After 10 min preincubation at 25°C, the reaction was started with 0.1 ml xanthine oxidase and incubation was performed for 20 min at 25°C. After addition of 0.2 mM CuCl_2_, the absorbance of the solution at 560 nm was measured. The activity of SOD required to inhibit the level of NBT reduction by 50% was defined as 1 unit of activity.

### Measurement of serum copper level

For serum collection, mice were anesthetized and their chests were cleaned with ethanol. Blood was obtained via closed cardiac puncture by means of a 22-guage hypodermic needle [25]. Blood from each group (CuNG treated and untreated; normal and EAC/Dox bearing mice) was taken in separate glass tubes, clotted, chilled to 4°C and centrifuged for 20 min at 3,000 rpm. Serum was removed, immediately filtered (0.22 *μm*) and stored at 4°C (if used within 24 h) or frozen. To 100 μl serum taken in a test tube, 3.9 ml of nitric acid (2.5%) was added and vortexed for 5 min. The solutions were kept at 37°C incubator for 6 h with occasional shaking. The mixture was centrifuged at 500 × g for 5 min. Cu was measured in the clear supernatant in Flame Atomic Absorption Spectrophotometer (AAS) [Varian Spectra 200 FS, hollow cathode lamp, Flame type: Air acetylene; replicate 3; wavelength 324.8 nm].

### Measurements of copper in liver tissue

Liver homogenate was prepared following the method of Hafemann *et al *[21]. To 100 μl homogenate taken in a test tube, 3.9 ml of nitric acid solution (2.5%) was added and vortexed for 5 min. The solutions were kept at 37°C incubator for 6 h with occasional shaking. The mixture was centrifuged at 500 × g for 5 min. Cu was measured in the clear supernatant by AAS.

### Measurement of bile copper level

For bile collection, mice were anesthetized; chests were cleaned with ethanol and opened. Whole gall bladder was removed and total bile of gall bladder was dissolved in 100 μl double distilled water. To 100 μl bile solution 3.9 ml of nitric acid solution (2.5%) was added and vortexed for 5 min. To avoid co-precipitation each sample was centrifuged at 500 × g at 37°C. Cu was measured in the clear supernatant by AAS.

### Measurements of copper in urine

Mice were fed with excess amount of water with the help of feeding needle and then urine was collected at different time intervals like 4 h, 24 h and 48 h after CuNG injection i.p. 100 μ*l *urine was added to 3.9 ml of nitric acid solution (2.5%) and vortexed for 2 min. To avoid co precipitation each sample was centrifuged at 500 × g at 37°C. Cu was measured in the clear supernatant by AAS.

### Measurements of copper in peritoneal fluid (PF)

Mice were anesthetized and peritoneal fluid was collected at different time intervals like 4 h, 24 h and 48 h after CuNG injection i.p. The maximum amount of peritoneal fluid collected at 4 h was 500 μ*l*; at 24 h and 48 h only 50 μl PF was collected. 50 μl PF was added to 1.95 ml of nitric acid solution (2.5%) and vortexed for 2 min. To avoid co precipitation each sample was centrifuged at 500 × g at 37°C. Cu was measured in the clear supernatant by AAS.

### Measurements of reactive oxygen species (ROS)

2 × 10^6 ^EAC cells from EAC/S bearing or EAC/Dox bearing (untreated or CuNG treated) mice or 10 mg of tissue (liver, lung, heart or kidney) in 1 ml of Hank's balanced salt solution (HBSS) were taken and NBT assay for ROS was performed according to Beauchamp and Fridovich [26] with minor modifications. Tissues were homogenized in tissue homogenizer. To each test sample, 0.5 ml of NBT-HBSS (1 mg NBT/ml) was added and incubated at 37°C for either 4 h (in case of EAC cells) or 8 h (in case of tissues). Then test samples were centrifuged and pellets were washed thrice with methanol. Following this the samples were dissolved in 1 ml 2 M KOH and 1 ml of DMSO and then OD_630 _was measured. OD values were compared with a standard curve constructed with NBT and ROS generation was expressed as μM NBT equivalent/2 × 10^6 ^cells or μM NBT equivalent/10 mg tissue.

### Detection of surface MRP1 expression by flowcytometry

For the determination of surface MRP1 expression, EAC/Dox cells were harvested from tumor-bearing mice and incubated in presence of anti-MRP1 polyclonal antibody following appropriate blocking. Cells were then washed and incubated with anti-rabbit IgG-FITC conjugated secondary antibody. Following washing, cells were fixed with paraformaldehyde and surface MRP1 expression was determined on FACS, fluorescence detector equipped with 488 nm argon laser light source and 623 nm band pass filter (linear scale) using CellQuest software (Becton Dickinson). Appropriate isotype control was taken in all cases. A total of 10000 events were acquired and analysis of flow cytometric data was performed using ModFit software. A histogram of FITC-fluorescence (x-axis) versus counts (y-axis) has been displayed.

### Detection of surface MRP1 expression by confocal microscopy

For the assessment of surface MRP1 expression, EAC/Dox cells were incubated in presence of anti-MRP1 polyclonal antibody following appropriate blocking. Cells were then washed and incubated with anti-rabbit IgG-FITC conjugated secondary antibody. Following washing, cells were fixed with paraformaldehyde and surface MRP1 expression was visualized using a confocal microscope (LSM 510; Carl Zeiss, Jena, Germany).

### Statistical Analysis

Each experiment was performed 3 to 5 times and results are expressed as mean ± SD or Student's t-test for significance was performed and p < 0.01 was considered significant. Flow cytometric and fluorescence microscopic data show representative data of at least three independent experiments.

## Results

### CuNG treatment in vivo could induce ROS generation by EAC/Dox cells and reduce their surface MRP1 expression

**Figure 1:**
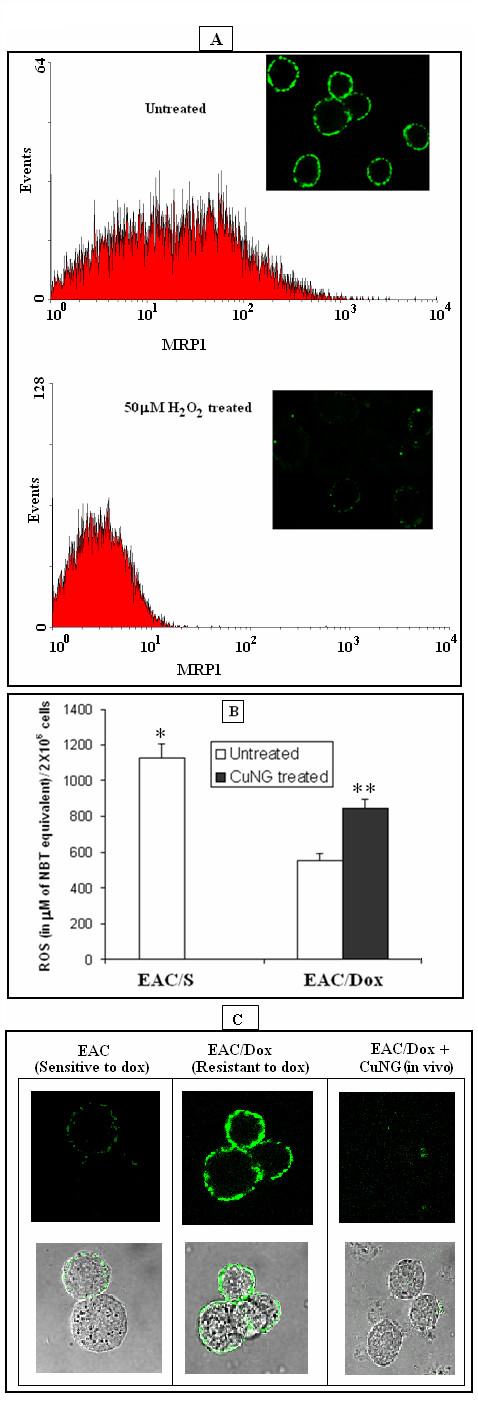
(A) Effect of exogenous H_2_O_2 _on MRP1 expression. EAC/Dox cells were incubated overnight in presence of 50 μM H_2_O_2_. Surface MRP1 expression was studied by flow cytometry and confocal microscopy. Representative data of 5 independent experiments is presented. (B) ROS generation by EAC/Dox cells ex vivo 24 h following in vivo CuNG treatment. CuNG treatment given after 7 days following inoculation with EAC/Dox cells. Results are compared with ex vivo ROS generation by EAC cells derived from EAC/S and EAC/Dox bearing mice. EAC/Dox cells following in vivo CuNG treatment and EAC/S cells generated significantly higher levels of ROS compared to EAC/Dox cells from untreated animals (*, P < 0.001 and **, P < 0.005). Results are presented as mean ± S.D. of 3 independent experiments. Each experiment was done with 5 animals in each experimental group. (C) Confocal microscopy of the effect of CuNG on MRP1 expression in EAC/Dox cells. EAC/Dox cells expressed higher levels of MRP1 than EAC/S cells and CuNG treatment reduced MRP1 expression. Results presented are representative of 3 independent experiments.

Increased ROS levels have been reported to reduce P-gp expression [6]. The EAC/Dox cells were shown to over express MRP1 [16] with respect to their sensitive counterpart. Interestingly, overnight incubation of EAC/Dox cells in presence of exogenous 50 μM H_2_O_2 _(which was non-toxic concentration as manifested by >98% viability) could also reduce surface MRP1 expression of these cells (Fig. 1A). Therefore, ROS also plays a crucial role for the maintenance of MDR phenotype in our cell model.

Since we have shown earlier that CuNG is a good resistance modifier [16] and as copper has been reported to induce apoptosis by generation of ROS [18], we tested whether CuNG treatment in vivo could also increase ROS generation in EAC/Dox cells. Interestingly, CuNG treatment in vivo was found to induce ROS generation in EAC/Dox cells by both NBT reduction (Fig. 1B) [>90% of the NBT reduction was inhibitable by SOD (data not shown)] as well as with H_2_O_2 _trapping dye, Dichlorofluorescein-diacetetate (DCF-DA) and Cytochrome c reduction (data not shown). This prompted us to investigate whether CuNG treatment could inhibit MRP1 expression. It was observed that in vivo CuNG treatment also decreased surface expression of MRP1 in EAC/Dox cells within 3 days (Fig. 1C). Interestingly, the surface MRP1 expression of EAC/Dox cells isolated from CuNG treated mice was even lower than that of EAC/S cells. This might be due to a contributory effect of NO that was also produced upto 72 h following CuNG treatment (data not shown). We have also found that H_2_O_2 _along with sodium nitroprusside caused a more profound down-regulation of MRP1 expression in EAC/Dox cells (data not shown).

### CuNG treatment in vivo reduces ROS generation in heart and lung in EAC/Dox bearing mice

Since increased ROS generation in different vital organs like heart, lung, liver and kidney may be toxic for these organs, we determined whether CuNG treatment also modulated ROS generation in these vital organs.

**Figure 2:**
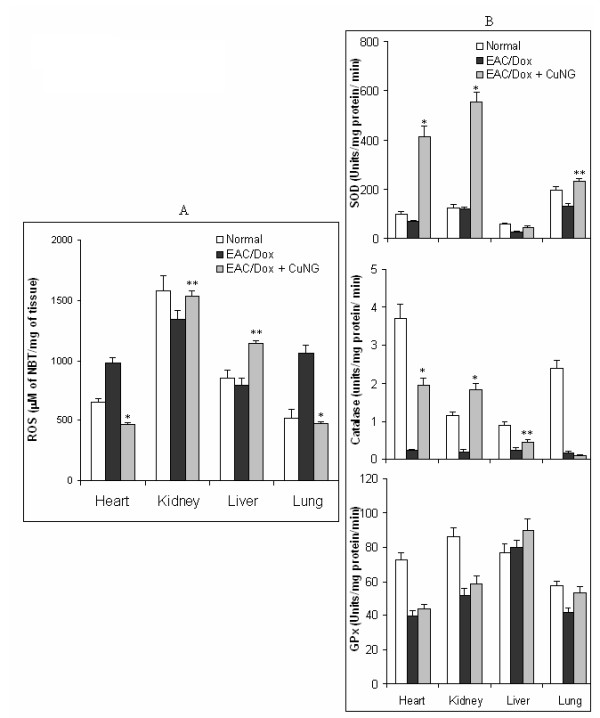
(A) Effect of in vivo CuNG treatment on ROS generation in heart, kidney, liver and lung of normal and EAC/Dox bearing mice. Results are presented as mean ± S.D. of 4 independent experiments, each involving 3 animals in each experimental group. Compared to untreated EAC/Dox bearing animals, In vivo CuNG treatment significantly increased ROS generation in kidney and liver, while decreased ROS generation in heart and lungs (*, P < 0.005 and **, P < 0.01). (B) Effect of in vivo CuNG treatment on SOD, catalase and GPx activity in heart, kidney, liver and lung of normal and EAC/Dox bearing mice. Results are presented as mean ± S.D. of 5 independent experiments, each involving 4 animals in each experimental group. Compared to untreated EAC/Dox bearing animals, In vivo CuNG treatment significantly increased SOD activity in heart, kidney and lungs, while increased catalase activity in heart, kidney and liver (*, P < 0.005 and **, P < 0.01).

As shown in Fig. 2A, CuNG treatment in vivo reduced ROS generation in heart and lung significantly (P < 0.01 and P < 0.001 respectively) in EAC/Dox bearing mice. Interestingly enough, CuNG treatment increased ROS generation in liver of these animals.

Since CuNG treatment in vivo modulated the ROS generation in vital organs, we studied the status of antioxidant enzymes related to phase II detoxification in these organs.

### Activities of SOD and CAT in heart, kidney, liver and lung in EAC/Dox bearing mice following CuNG treatment in vivo

The activity of SOD decreased in heart (~35%), kidney (~5%) liver (~50%) and lung (~20%) (Fig. 2B upper panel) in EAC/Dox bearing mice compared to their normal counterparts. Treatment with CuNG increased the activity of SOD in heart by ~3 folds, in kidney by ~2.5 folds, in liver by 80% and in lung by ~35% with respect to untreated EAC/Dox bearing mice.

CAT activity decreased in all vital organs like heart, kidney, liver and lung in EAC/Dox bearing mice in comparison to normal mice (Fig. 2B, middle panel). Treatment of CuNG in EAC/Dox bearing mice tremendously increased CAT activity in heart (~5.5 folds) and kidney (~5 folds) but the same was increased only ~30% in liver. Interestingly, the CAT activity of lung remained almost unchanged following CuNG treatment.

GPx activity was observed to be low in heart, kidney, and lung of EAC/Dox bearing mice compared to normal mice (Fig. 2B, lower panel). In EAC/Dox bearing mice CuNG increased GPx activity only slightly in heart, kidney and liver but moderately in lung (~30%) at 24 h post-treatment.

Interestingly, the SOD activity of EAC/Dox cells (which was observed to be slightly higher in these drug resistant cells compared to their sensitive counterpart) was observed to decrease by ~15% within 24 h following in vivo CuNG treatment (data not shown). Although the catalase activity remained more or less unchanged, the GPx activity also increased to some extent in tumor cells following CuNG treatment of EAC/Dox bearing mice (data not shown).

### CuNG treatment temporarily alters levels of GSH in heart, kidney, liver and lung of EAC/Dox bearing mice

**Figure 3:**
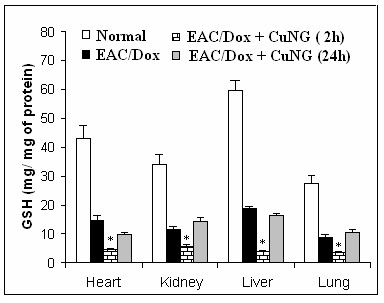
Effect of in vivo CuNG treatment on GSH levels 2 h and 24 h post-treatment in heart, kidney, liver and lung of EAC/Dox bearing mice. Results are compared with corresponding controls. Results presented as mean ± S.D. of 3 independent experiments, each involving 5 animals in each experimental group. GSH levels decreased significantly in all organs 2 h following CuNG treatment in vivo in EAC/Dox bearing animals compared to their untreated counterparts (*, P < 0.01).

As GSH is important for scavenging ROS and maintaining a reducing environment with in the cell, and since CuNG depletes GSH in EAC/Dox cells, we have studied whether CuNG treatment also lowers the GSH levels in heart, lung, liver and kidney. GSH levels were low in these organs of EAC/Dox bearing mice compared to that in their normal counterparts. Although CuNG treatment depleted GSH in all the organs tested by 2 h, the levels were restored by 24 h post treatment (Fig. 3), indicating that CuNG only temporarily depleted GSH levels in these organs.

### Status of liver and renal function following CuNG treatment

Since CuNG treatment increased ROS generation in liver of EAC/Dox bearing mice, we tested different parameters of liver and renal function to decipher whether there was any apparent hepatic or renal toxicity following treatment with CuNG.

As shown in Table 1, the levels of serum urea, serum glutamate pyruvate transaminase (SGPT/ALT) and serum glutamate oxaloacetate transaminase (SGOT/AST) levels drastically decreased in EAC/Dox bearing mice in comparison to their normal counterparts. CuNG treatment completely restored the SGOT level and partially restored the SGPT and urea levels in serum within 24 h (Table 1). Alkaline phosphatase (ALKP) levels in serum were comparable in normal and EAC/Dox bearing mice. CuNG treatment did not significantly alter serum ALKP level in EAC/Dox bearing mice.

### Excretion of copper following CuNG treatment

Accumulation of excess copper is known to be toxic. Therefore, we were interested to know whether copper was excreted or not following CuNG treatment.

**Figure 4:**
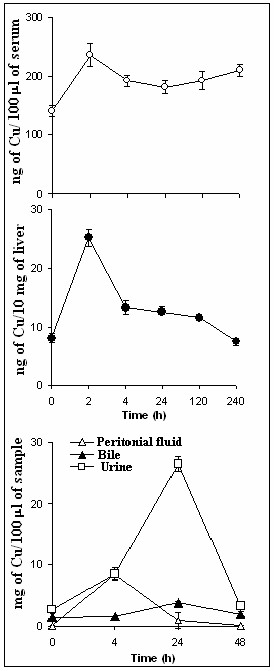
Copper levels in serum, liver and in peritoneal fluid, bile and urine of normal and EAC/Dox bearing mice following CuNG treatment. Results presented as mean ± S.D. of 6 independent experiments, each involving 3 animals in each experimental group.

After CuNG injection, released copper was measured in bile, urine and in peritoneal fluid by AAS. Each mouse was injected 180 μl solution of CuNG (1 mg/ml) i.p. that is equivalent to 36.82 μg of Cu. Very high amount of copper was released through urine (26.39 ± 1.6 μg) within 24 h. The amount of copper excreted in bile was lower than that of the urine (Fig. 4). We were able to collect 400 μl of peritoneal fluids within 2 to 4 h of CuNG injection, but after 24 h we collected only 25 μl peritoneal fluid. In CuNG treated Swiss albino mice (6 week old, 18–22 gm weight) the amount of urine collected was only 400 ± 23 μl/day. After 24 h of CuNG injection, the volume of collected urine reached the normal level i.e., 800–1000 μl/day. High amounts of copper were excreted through bile within 24 h of CuNG injection but reached almost to the normal level within 48 h (Fig. 4) is indicative that CuNG is metabolized in the liver and excreted through bile [27].

## Discussion

ROS have multiple functions [1] and are implicated in tumor initiation and progression [28-30] as well as in induction of apoptosis of various cancer cells, including drug resistant cancers [2-4]. Often, the ability of a therapeutic agent to induce apoptosis of cancer cells depends upon the ability of those cancer cells to generate ROS [5]. Drug resistant cancers often show very low levels of ROS. This is usually due to high intracellular GSH level [7] and enhanced activities of antioxidant enzymes [8]. In the present investigation, we have also found that the level of ROS generation is lowered in EAC/Dox cells, which contain high levels of intracellular GSH [16], compared to their drug sensitive counterparts. Moreover, low levels of ROS favor the expression of ABC transporters like P-gp [6] on cancer cell surface. We have also found that an oxidative environment reduced MRP1 expression on EAC/Dox cells. Interestingly, CuNG treatment, which resulted in elevated ROS generation by EAC/Dox cells, could also reduce their surface MRP1 expression. Elevation of ROS generation by EAC/Dox cells following CuNG treatment was quite expected as CuNG can deplete GSH that can actively scavenge ROS [15,16]. The effect of oxidative stress on P-gp has been well reported previously [6] but the phenomenon of ROS mediated (as well as NO mediated) down regulation of MRP1 expression, which has been reported here, is less known. This has important implication in therapy. It was found that by suppressing surface MRP1 expression, CuNG converted EAC/Dox cells susceptible to dox induced killing. These cells were now observed to accumulate dox and undergo apoptosis (data not shown).

Although ROS is important for cell signaling and different physiological processes [31-33], too much ROS level and oxidative stress is harmful [34,35]. Since CuNG could elevate ROS in EAC/Dox cells, we undertook further studies on levels of ROS generation, activation status of different antioxidant enzymes and GSH levels in different vital organs like heart, lung, liver and kidney following CuNG treatment. We observed that ROS levels increased in heart and lung but not in liver and kidney in EAC/Dox bearing mice compared to their normal counterparts. Interestingly, CuNG treatment reduced ROS in heart and lung but not in liver and kidney in EAC/Dox bearing mice. It has been reported earlier that SOD is decreased in lung and liver of cancer patients [36]. We have also observed a decrease in SOD activity in heart, liver and lung, but not in kidney of EAC/Dox bearing mice compared to their normal counterparts. This might reflect stress following EAC/Dox inoculation and body's response to try and increase ROS generation. CuNG treatment strongly increased SOD activity in all organs tested in EAC/Dox bearing mice. On the other hand, CAT activity was strongly increased in heart and kidney while the increase in CAT activity in liver was only marginal in response to CuNG treatment. Moreover, CuNG treatment caused a significant (P < 0.05) increase of GPx activity in lung. Thus oxidative stress is well countered by increase in SOD and CAT activities in heart and kidney and by SOD and GPx in lung following CuNG treatment in EAC/Dox bearing mice. Furthermore, these data indicate that the ROS generating effect of CuNG might be masked by increased SOD and CAT activity in different organs. Interestingly, CuNG treatment could elevate ROS generation to high levels in tumor tissues that was not observed in normal tissues of cancer bearing mice. However, since CuNG could efficiently depleted liver-GSH, an increase of ROS in liver was expected. Indeed this was found in the present investigation. This in turn might actually cause an oxidative environment in the organ along with increased CAT and GPx activities with detrimental effects to tumor cells [6,8,37]. Decreased levels of SGPT (ALT), SGOT (AST) and urea in EAC/Dox bearing mice may be indicative for ensuing hepatic failure. CuNG treatment brought back serum SGPT and SGOT to near normal values and also elevated serum urea level to some extent indicating that no hepatic damage may have occurred due to CuNG induced oxidative stress in liver. Moreover, CuNG did not significantly alter ROS generation in heart and decreased ROS generation in kidney, although it increased ROS generation in lung and liver of normal animals (data not shown). Of course, the effect of CuNG on different organs of normal animals is not a good indicator of its utility as copper homeostasis is disturbed in drug resistant cancer bearing animals [38]. Interestingly, CuNG was found to have no suppressive effect on lymphoproliferation (data not shown) and was rather found to reduce tumor-induced immunosuppression [17].

Copper accumulation may lead to toxicity [39]. Since CuNG is a copper complex, it increases the level of Cu in the system. It has been reported that serum copper level was elevated in animals and humans with cancer [38,40]. When CuNG was injected in EAC/Dox bearing mice, the concentration of copper in serum and liver was also increased and found to be highest at 2 h. As copper is metabolized in liver [27] we studied its level in liver and found that copper level in liver increased till 2 h following CuNG injection and then decreased. The increase of liver Cu was found to be inversely proportional to liver-GSH level; the level of GSH has been found to be depleted by 50% after the injection of CuNG. Depletion of GSH by Cu has previously been reported e.g., by CuSO_4 _[41,42]. Cu is stored mostly as metallothionein (MT)-copper complexes in the organism while the amounts of unbound free Cu is almost negligible; upon copper injection, GSH binds to Cu before the metal complexes with MT [41]. In the present report the initial depletion of GSH with increase in Cu level in the liver induced by CuNG may be due to the formation of GS-CuNG complexes [16]. We had reported some data on GS-CuNG conjugate [16]. The level of copper rises initially in EAC/Dox cells following CuNG treatment, which again quickly decreased within 2–3 h due to expulsion of GS-CuNG conjugate [16]. The detailed works on the structural aspect of GS-CuNG conjugate are being pursued. Conjugation with GSH is a well-known mechanism of exclusion of xenobiotics from cells. After CuNG administration most of the copper (serum-Cu and liver-Cu) was eliminated from the system by 24 h and some amount of copper might be stored in the system (liver) possibly through the formation of Cu (I)-metallothionein complex [41]. Similarly, copper level is increased in different tissues initially after CuNG treatment, which is decreased within 24 h (data not shown). We have utilized the depletion of GSH to sensitize drug resistant cancer cells with elevated GSH level to anticancer drugs [16,43]. Moreover, CuNG acts as immunomodulator at a lower dose and releases cytokines and overcomes drug resistance [17]. We have also observed that CuNG down-regulates the phosphorylation status of signaling molecules like Akt, which is related to drug resistance and cell-survival in EAC/Dox cells, as evidenced from the observation that wortmanin pretreatment in vitro could reduce MRP1 expression and sensitize drug resistant cells (EAC/Dox) towards doxorubicin (unpublished data).

## Conclusion

Thus, we conclude that besides reversing resistance toward dox by depletion of GSH [16], CuNG could also induce ROS generation and down regulation of surface MRP1 expression in EAC/Dox cells. The depletion of GSH following formation and expulsion of GS-CuNG conjugate might be partially responsible for elevation of ROS in EAC/Dox cells. Interestingly, neither CuSO_4 _nor the organic backbone (NG) could reduce surface MRP1 expression of the EAC/Dox cells. Moreover, CuNG could modulate SOD, CAT and GPx to reduce oxidative stress in heart and lung. CuNG treatment of EAC/Dox bearing mice also restored SGPT and SGOT levels to almost normal values and thereby rescued them from liver failure. Thus CuNG is not only a resistance modifier but it also possesses tissue protective activity.

## Competing interests

The author(s) declare that they have no competing interests.

## Authors' contributions

AM designed and performed some major biochemical experiments, did the confocal microscopy and drafted the manuscript; JMB participated in designing and performing some major biochemical experiments, did the flowcytometry and also participated in drafting the manuscript; SM, SC, GSP and PD performed biochemical experiments; SP performed all the hepatic and renal function tests; PM maintained the cell lines; TE advised and designed some biochemical experiments and participated in drafting the manuscript; all experiments were performed in the laboratories of SR and SKC under their guidance. All authors have read and approved the final manuscript.

## Pre-publication history

The pre-publication history for this paper can be accessed here:



## Figures and Tables

**Table 1:** Effect of CuNG on hepatic and renal functions of EAC/Dox bearing mice

	Urea (mg/dL)	ALKP (U/L)	SGOT (U/L)	SGPT (U/L)
EAC/Dox-bearing	25 ± 1.5	185 ± 5.5	174 ± 7.5	65 ± 6.0
EAC/Dox-bearing +CuNG (ip)	32 ± 2.1	181 ± 4.5	471 ± 12.5	96 ± 7.5
Normal	52 ± 3.5	182 ± 4.0	422 ± 11.5	120 ± 9.5
